# Nasal carriage of methicillin resistant *Staphylococcus aureus* among health care workers at Al Shifa hospital in Gaza Strip

**DOI:** 10.1186/s12879-016-2139-1

**Published:** 2017-01-05

**Authors:** Nabil Abdullah El Aila, Nahed Ali Al Laham, Basim Mohammad Ayesh

**Affiliations:** 1 Medical Laboratory Sciences department, Faculty of Applied Sciences, Al Aqsa University, P.O. box 4051, Gaza, Palestine Israel; 2 Department of Laboratory Medicine, Faculty of Applied Medical Sciences, Al Azhar University, P.O. Box 1277, Gaza, Palestine Israel

**Keywords:** MRSA, Healthcare workers, Nasal carriage, Gaza Strip

## Abstract

**Background:**

Nasal carriage of *Staphylococcus aureus* among hospital personnel is a common cause of hospital acquired infections. Emergence of drug resistant strains especially methicillin resistant *S. aureus* (MRSA) is a serious problem in hospital environment. Therefore, the aim of this study was to determine the nasal carriage rate of *S. aureus* and MRSA among Health Care Workers (HCWs) at Al Shifa Hospital, the major hospital in Gaza Strip.

**Methods:**

A cross sectional study was conducted on 200 HCWs. Nasal swabs were collected during February — April 2015, and cultured on blood and mannitol salt agar. The isolates were identified as *S. aureus* based on morphology, coagulase test, DNase test and mannitol salt agar fermentation. Disk diffusion antibiotic susceptibility tests were performed according to the guidelines of the Clinical and Laboratory Standards Institute. MRSA were confirmed by detection of the *mecA* gene by PCR.

**Results:**

Out of the 200 healthcare workers, 62 (31%) carried *S. aureus,* of which 51 (82.3%) were MRSA. Therefore, 25.5% of all HCWs were identified as MRSA carriers. MRSA carriage rate was highest among nurses (30.4%) whereas the carriage rate among doctors was (16%). The majority of MRSA carriers were workers of internal medicine department and surgical wards (41.3 and 35% respectively). Out of the 51 MRSA isolates identified by oxacillin disc resistance, 40 were confirmed by PCR targeting the *mecA* gene. Penicillin showed the highest rate of resistance among MRSA and MSSA isolates (100%).

**Conclusions:**

The high rate of nasal MRSA carriage among healthcare workers found in this study is alarming and highlights the need for adjusted infection control measures to prevent MRSA transmission from HCWs to the vulnerable patient.

## Background

MRSA is a major nosocomial pathogen that causes severe morbidity and mortality worldwide [1]. It has emerged as one of the commonest causes of hospital acquired infection and continues to remain an important factor contributing to failure of management [2].

Nasal carriage of *S. aureus* appears to play a key role in the epidemiology and pathogenesis of infection [3]. HCWs who are at interface between the hospital and the community may serve as agents of cross contamination of hospital acquired and community acquired MRSA [4].

Knowledge of the prevalence of MRSA and its antimicrobial profile is necessary for selection of the appropriate empirical antimicrobial treatment for *S. aureus* infections [5]. In particular, screening for and eradication of MRSA from colonized HCWs have been recognized and recommended as an important part of a comprehensive infection control policy for this organism.

In 2007, a Mediterranean study found that the highest proportions of MRSA were reported by Egypt (52%), Cyprus (55%), Algeria (45%), Malta (50%) and Jordan (56%), in comparison to other Mediterranean countries such as Lebanon (12%), Tunisia (18%) and Morocco (19%) [6]. However, few reports are available about the epidemiology of *S. aureus* and MRSA in HCWs in Palestine. Several studies reported the prevalence of *S. aureus* in Palestine on a hospital— and community-acquired basis [7–12]. Al Laham et al recently reported MRSA carriage rate of 22.6% among HCWs in three different hospitals in Gaza Strip [13].

In this follow up study we report the prevalence of *S. aureus* and MRSA carriage in a larger group of HCWs from Al Shifa hospital with emphasis on its distribution based on different health care professions. Furthermore, we describe the antibiotic susceptibility patterns of the *S. aureus* and MRSA isolates. The outcomes of the study are useful for formulating a MRSA infection control policy in hospitals of Gaza strip.

## Methods

### Study design

A cross sectional study involving 200 health care personnel who are working at different departments in Al Shifa hospital during the period from February to April 2015.

HCWs with a history of upper respiratory tract infection, fever, recent nasal surgery, diabetes, use of nasal medications or antimicrobial therapy and immunocompromised patients were excluded.

The study was approved by the Ministry of Health, and an informed consent was obtained from each participant.

### Collection and culture of specimens

Nasal swabs were collected from the anterior nares of the HCWs using a cotton swab in AMIES transport medium.

The swab was inserted into each nostril in turn to a depth of approximately one cm and rotated 4–5 times both clockwise and counterclockwise. The swabs were immediately transported to the microbiology laboratory for further processing. Specimens were inoculated onto Blood agar and incubated overnight at 37 °C. Suspected colonies of *S. aureus* were confirmed by morphology, Gram stain, catalase test, fermentation of Mannitol salt agar, DNAse production on DNA agar and coagulase tests.

Antibiotic susceptibility testing of all isolates was performed by modified Kirby Bauer disc diffusion method as recommended by CLSI guidelines [14] using Mueller-Hinton agar. The antibiotics used in this study were penicillin (10 Units), amoxicillin-calvulanic (30 μg) acid, erythromycin (15 μg), vancomycin (30 μg), tetracycline (30 μg), cefuroxime (30 μg), gentamycin (10 μg), rifampicin (5 μg), clindamycin (2 μg), and ciprofloxacin (5 μg) (Himedia - India). Methicillin resistance was detected using oxacillin (1 μg) disc diffusion test. Briefly, single isolated colonies were selected and inoculated in Mueller-Hinton broth until its turbidity is comparable to 0.5 McFarland turbidity standard. Then the plates were inoculated with each broth culture and left to dry at room temperature before the application of antibiotic discs. The plates were incubated at 35 ± 2 °C in ambient air for 16–18 h. Plates with oxacillin were incubated at 33–35 °C because testing at temperatures above 35 °C may not detect MRSA as recommended by CLSI guidelines [14]. Zone of inhibition for each antimicrobial agent was interpreted, reporting the organism as resistant, intermediate or susceptible. *S. aureus* ATCC 33,592 (*mec*A positive, resistant) and *S. aureus* ATCC 29,213 (*mec*A negative, susceptible) were used as quality control strains.

### DNA extraction from isolates

DNA was extracted from cultured isolates by alkaline lysis as previously described [15]. Briefly, one bacterial colony was suspended in 20 μl of lysis buffer (0.25% sodium dodecyl sulfate, 0.05 N NaOH) and heated at 95 °C for 15 min. The cell lysate was diluted by 180 μl of distilled water. The cell debris was pelted by centrifugation at 16,000 x*g* for 5 min. and the supernatants were used for PCR or frozen at −20 °C until further use.

### Detection of *mecA* gene by PCR

The *mecA* gene and *femA* endogenous control gene were simultaneously amplified in the same reaction. The primers used to amplify the *mecA* gene were (forward: 5’- AAGCGACTTCATCTATTAGGTTAT-3’ and reverse: 5’-TATATTCTTCGTTACTCATGCCATAC-3’; [16]. The primers used to amplify the *femA* gene were (forward: 5’-AACTGTTGGCCACTATGAGT-3’ and reverse: 5’-CCAGCATTACCTGTAATCTCG-3’; [17]. The reactions were performed in 25 μl final volumes in the presence of 1 μM of each primer, 2 μl DNA and 1X of the GoTaq Green MMX (Promega, USA).

The thermal cycling program for detection of both genes was as follows: one cycle of initial denaturation at 95 °C for 5 min; 34 cycles of denaturation at 95 °C for 30s, annealing at 60 °C for 30s, and extension at 72 °C for 1 min followed by a final extension at 72 °C for 5 min. The amplified products (*femA*: 306 bp and *mecA*: 402 bp) were resolved on a 2% agarose gel. The fragments were stained with ethidium bromide and visualized and photographed using gel documentation system. A 100 bp ladder was run as a molecular weight marker.

Another set of primers targeting the *mecA* gene was used (forward: 5’- AAAATCGATGGTAAAGGTTGGC-3’ and reverse: 5’-GTTCTGCAGTACCGGATTTGC-3’) for confirmation of *mecA* gene expression using the reaction components described above [18]. After initial denaturation at 95 °C for 1 min, the samples were subjected to 35 cycles of denaturation at 95 °C for 1 min, annealing at 54 °C for 1 min and extension at 72 °C for 1 min. A final extension was performed at 72 °C for 5 min. The expected amplicon size is 533 bp.

Positive and negative controls were added in each run, we used reference strains that are mecA positive and negative. *S. aureus* ATCC 33,592 (positive control for *mec* gene, MRSA); *S. aureus* ATCC 29,213 negative control.

### Statistical analysis

The results were tabulated and analyzed using version 20 of the Statistical Package for the Social Sciences (SPSS). Frequencies, cross tabulation and appropriate statistical tests as Chi-square test and fisher exact test were performed. A *P-*value of less than 0.05 was considered significant.

## Results

**Fig. 1:**
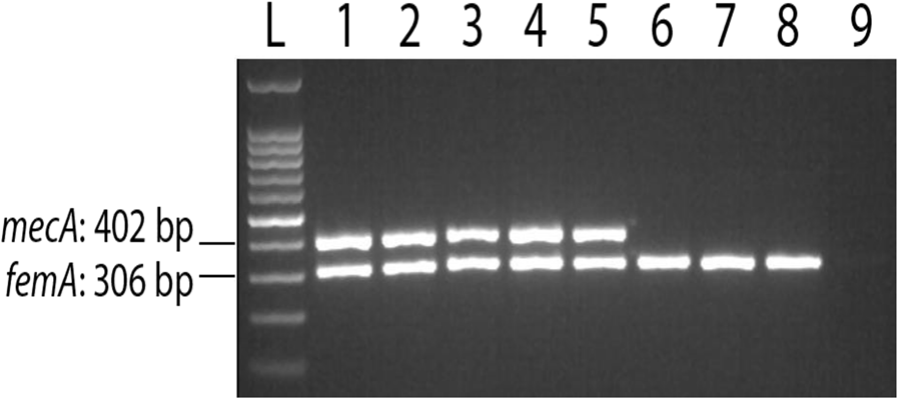
A representative result of *mecA* and *femA* PCR. Lane L: 100 bp DNA ladder; lane 1: positive control; lanes 2, 3, 4 and 5 are tested isolates with positively amplified *mecA* and *femA* genes; lanes 6, 7 and 8 are negative for the *mecA* gene and positive for the *femA* gene; lane 9 is a blank

Overall, 62 bacterial isolates were phenotypically identified as *S. aureus* as described in the materials and methods. All of the 62 isolates were confirmed as *S. aureus* by targeting the *femA* gene. Accordingly, the rate of nasal carriage of *S. aureus* among HCWs is 31% (62/200). Among them 51 (82.3%) were identified as MRSA using the oxacillin disc resistance (≤11 mm; Table 1). However, using PCR targeting the *mecA* gene in *S. aureus*, only 40 isolates were confirmed as MRSA (Fig. 1).

**Table 1:** Distribution of *S. aureus* and MRSA carriage among the different health professions

Health workers	No. of samples	*S. aureus* %(62)	MRSA %(51)	MSSA %(11)	*P*. Value
Doctor	50	14(28%)	8(16%)	6(12%)	0.058
Nurses	125	42(33.6%)	38(30.4%)	4(3.2%)	
Pharmacist	6	2(33.3%)	2(33.3%)	0	
Radiologist	8	1(12.5%)	1(12.5%)	0	
Technicians	11	3(27%)	2(18%)	1(9%)	
Total	200	62(31%)	51(25.5%)	11(5.5%)	

*Legend*: *MRSA* Methicillin resistant *Staph aureus*, *MSSA* Methicillin susceptible *Staph aureus*

*P* value <0.05 statistically significant

The overall nasal carriage rate of MRSA was 25.5% (51/200). *S. aureus* carriage rate was highest among nurses 33.6% (42/125), whereas carriage among doctors was 28% (14/50).

MRSA carriage rate was significantly highest (*P* = 0.001) among nurses 30.4% (38/125) compared to other professions (Table 1). Moreover, 90% (38/42) of the isolates of *S. aureus* carried by the nurses were MRSA. The carriage rate among doctors was 16% (8/50).

The highest rate of *S. aureus* carriage was found in HCWs of the internal medicine and emergency departments (44.8 and 44.4% respectively). On the other hand, the highest MRSA carriage rate was found in internal medicine and surgery wards (41.3 and 35% respectively; Table 2). All of the 17 *S. aureus* isolates collected from healthcare workers of the surgery department were MRSA (100%). Additionally, 12 out of the 13 *S. aureus* isolates obtained from healthcare workers of the internal medicine department (92%) were MRSA (Table 2).

**Table 2:** Distribution of *S. aureus* and MRSA isolates by wards/departments

Wards/	No of samples	*S. aureus* (%)	MRSA (%)	MSSA (%)	*P*. Value
Department	(*n* = 200)	(*n* = 62)	(*n* = 51)	(*n* = 11)	
Surgery	48	17(35%)	17(35%)	0	0.019
Internal medicine	29	13(44.8%)	12(41.3%)	1(3.4%)	
Emergency	27	12(44.44%)	8(25.9%)	4(14.8%)	
Urology	11	1(9%)	0	1(9%)	
Burn department	10	2(20%)	2(20%)	0	
I.C.U	18	6(33.3%)	5(27.7%)	1(5.5%)	
Pharmacy	6	2(33.3%)	2(33.3%)	0	
X-ray	8	1(12.5%)	1(12.5%)	0	
Laboratory	11	3(27%)	2(18%)	1(9%)	
Chest department	6	1(16.6%)	1(16.6%)	0	
Outpatient clinic	7	2(28.5%)	1(14.25%)	1(14.25%)	
Maternity	13	1(7.7%)	1(7.7%)	0	
Ear, nose and throat	6	2(33.3%)	0	2(33.3%)	

*Legend*: *MRSA* Methicillin resistant *Staph aureus*, *MSSA* Methicillin susceptible *Staph aureus*

*P* value <0.05 statistically significant

All *S. aureus* isolates (100%) were resistant to penicillin. However, sensitivity was high, but with variable degrees, to gentamicin, ciprofloxacin, rifampicin, clindamycin and vancomycin (96.7, 90.3, 88.7, 88.7, and 85.4% respectively; Table 3).

**Table 3:** Antibiotic susceptibility pattern of *S. aureus* isolates

Antibiotics	Susceptible (%)	Intermediate (%)	Resistant (%)
Oxacillin	11 (17.7%)	0 (0%)	51 (82.3%)
Penicillin	0 (0%)	0 (0%)	62 (100%)
Amoxicillin/clavulanic acid	30 (48.4%)	1 (1.6%)	31 (50.0%)
Erythromycin	44 (80.0%)	7 (11.3%)	11 (17.8%)
Vancomycin	53 (85.5%)	0	9 (14.5%)
Tetracycline	53 (85.8%)	6 (9.8%)	3 (4.8%)
Cefuroxime	53 (85.8%)	6 (9.8%)	3 (4.8%)
Gentamycin	60 (96.8%)	1 (1.6%)	1 (1.6%)
Rifampicin	55 (88.7%)	4 (6.4%)	3 (4.8%)
Clindamycin	55 (88.7%)	5 (8.0%)	2 (3.2%)
Ciprofloxacin	56 (90.3%)	4 (6.4%)	2 (3.2%)

Similarly, high rate of resistance of MRSA isolates against penicillin were noticed (100%). On the other hand sensitivity of the MRSA isolates to gentamicin, ciprofloxacin, rifampicin, clindamycin, tetracycline and vancomycin was high (92.2, 88.2, 88.2, 86.3, 86.3 and 84.3%; Table 4). All MSSA isolates were suceptible to gentamicin, cefuroxime, ciprofloxacin, rifampicin and clindamycin (Table 4).

**Table 4:** Antibiotic susceptibility pattern of MRSA and MSSA isolates

Antibiotic	MRSA (*n* = 51), *n* (%)			MSSA (*n* = 11), *n* (%)		
	S	I	R	S	I	R
Oxacillin	0	0	51 (100%)	11 (100%)	0	0
Penicillin	0	0	51 (100%)	0	0	11 (100%)
Amoxicillin/ clavulanic acid	29 (56.9%)	0	22 (43.1%)	8 (72.7%)	0	3 (27.3%)
Erythromycin	33 (64.7%)	8 (15.7%)	10 (19.6%)	10 (90.9%)	0	1 (9.1%)
Vancomycin	43 (84.3%)	0	8 (15.7%)	10 (90.9%)	0	1 (9.1%)
Tetracycline	44 (86.3%)	2 (3.9%)	5 (9.8%)	9 (81.8%)	1 (9.1%)	1 (9.1%)
Cefuroxime	42 (82.4%)	6 (11.8%)	3 (5.9%)	11 (100%)	0	0
Gentamycin	47 (92.2%)	2 (3.9%)	2 (3.9%)	11 (100%)	0	0
Rifampicin	45 (88.2%)	3 (5.9%)	3 (5.88%)	11 (100%)	0	0
Clindamycin	44 (86.3)	5 (9.8%)	2 (3.92%)	11 (100%)	0	0
Ciprofloxacin	45 (88.2%)	4 (7.8%)	2 (3.92%)	11 (100%)	0	0

*Legend*: *MRSA* Methicillin resistant *Staph aureus*, *MSSA* Methicillin suceptible *Staph aureus*

## Discussion

Detection of MRSA nasal carriage among HCWs in the hospital is necessary particularly for those working in the critical care areas. These individuals act as a potential source of infection to their immunocompromised patients, resulting in their extended stay in the hospital [19].

Health care workers (HCWs) are at the interface between hospitals and communities. The survey for MRSA carriage among HCWs has mostly been conducted to investigate outbreaks or endemics but not in non-outbreak situations [4]. For instance, the substantial proportion of children in Taiwan with nasal MRSA colonization encouraged Huang et al to study the carriage rate of MRSA among pediatricians who cared for these children in non-outbreak situations. Around seven percent of pediatricians in Taiwan harbored CA-MRSA in their nares [20].

Recently, a community-based nasal carriage among healthy children and a hospital-based studies addressed the epidemiology of *S. aureus* and MRSA in Gaza Strip for the first time [9, 10] (Al Laham et al, 2015; Biber et al. 2012). However, data regarding the epidemiology of *S. aureus* and MRSA among HCWs in Gaza are very scant. To the best of our knowledge, our study was the second to address this topic.

In our study, the overall nasal carriage rate of *S. aureus* and MRSA was 31 and 25.5% respectively. Our results are comparable to those of a recent study that covered HCWs of three different hospitals in Gaza Strip and found a carriage rate of 42.1% for *S. aureus* and 22.6% for MRSA [13]. Slight variations were found between the two previous studies. In our study, nasal swabs were collected from 200 health care personnel who are working at different departments in Al Shifa hospital whereas in Al Laham study, the nasal swabs were collected from 140 HCWs from three different hospitals in Gaza. Moreover, in our study we have determined the rate of MRSA nasal carriage according to the professions among HCWs.

Our findings of *S. aureus* colonization among HCWs are also comparable to the findings of other studies in developing countries like Iran (31%; [21], Libya (39%; [22], Pakistan (48%; [23] and India (50%; [24], or in developed countries like Germany (33.8%; [25] and USA (30, and 43.8%; [26, 27].

The estimated prevalence of MRSA in our study was higher than that reported by studies conducted in Nepal (3.4%; [28], Ethiopia (12.7%; [29], Iran (5.3 and 3.4%; [21, 28], and Bangalore (8%; [19]. Our reported rate of *S. aureus* colonization is also higher than that reported by other studies conducted in Arabic countries and other developing countries like Libya (12.4%; [30], India (17.5%; [31], West Bank of Palestine (20.8%; [11] and Kuwait (21%; [32].

Differences in the prevalence of nasal carriage of *S. aureus* strains between countries and hospitals may be explained in part by differences in the quality and size of samples, the use of different microbiological methods (from sampling technique to culture media) and different interpretation guidelines. Moreover, different levels of commitment to infection control measures may contribute to these differences.

The frequency of MRSA carriage varies between hospital wards. Our results showed that that 76% of the MRSA carriers were working in the surgery and internal medicine wards. All *S. aureus* isolates recovered from the HCW of the surgery wards were MRSA whereas 12 out of 13 *S. aureus* isolates from the internal medicine were MRSA. These two departments are well known for their particularly high workload, crowdedness and relative shortage of HCWs.

Eradication of MRSA among the HCWs as well as improving the infection control measures may help limiting nosocomial transmission [33] although decolonization of patients have recently been shown to be more effective in high-endemicity settings [34].

Similarly, Khanel et al reported the HCWs from the surgical wards and operating rooms accounted for 28.6% of the MRSA carriers in Nepal [28].

In our study, the MRSA carriage was particularly high among the nurses (30.4%), followed by doctors (16%). This finding could be explained by the increased physical contact of nurses and doctors with patients and overcrowding in surgery departments at Al Shifa hospital which is a referral main tertiary hospital in the Gaza Strip.

In concordance with our study, Shibabaw et al reported the MRSA carriage was particularly high among nurses (21.2%), doctors (12.5%) and laboratory technicians (12.5%) [29]. In addition, in Nepal, MRSA carriage was highest among nurses (7.8%) [28].

Recently a number of studies proposed using cefoxitin to be superior in predicting the presence of *mecA* in *S. aureus* and coagulase negative *Staphylococci* with a high degree of sensitivity and specificity [35–37]. Cefoxitin was suggested to be used as a surrogate for the oxacillin disk diffusion test. In particular, cefoxitin would overcome the failure of routine oxacillin disk diffusion tests to detect heterogeneous MRSA populations, it is a good inductor of penicillin binding protein 2a production in *S. aureus* isolates that carry the mecA gene [36].

Out of 51 MRSA isolates detected by oxacillin diffusion test, only 40 were confirmed by PCR. In agreement with our results, the absence of *mecA* gene within resistant staphylococcal isolates was reported worldwide [38–40]. For example, Elhassan et al reported twelve out of the 123 MRSA isolates (9.8%) were *mecA* negative when using PCR targeting *mecA* gene [41]. Inconsistency between phenotypic and genotypic findings may result from mutations in the primers binding sites within the gene. For this purpose we have used a different set of primers to target the *mecA* gene [18]. However, the same results were obtained. This may also be attributed to intrinsic factors that compete with *mecA* gene in producing resistance phenomenon in regions with high prevalence of MRSA. Olayinka et al reported the complete absence of five major *SCCmec* types and *mecA* genes as well as the gene product of Penicillin binding protein 2a (PBP2a) in isolates which were phenotypically MRSA suggesting a probability of hyperproduction of β- lactamase as a cause of this phenomenon [42]. The possible existence of mechanisms other than presence of *mecA* gene requires further investigation.

Our and others findings undermine the reliability of *mecA* gene PCR in characterization of MRSA isolates, a point that should be under consideration by regional and reference laboratories.

In this study, the antibiogram profile of all *S. aureus* and MRSA isolates showed 100% resistance to penicillin. This pattern of resistance was shown in previous studies from Gaza Strip and the West Bank [8, 9, 11] and was also reported by others elsewhere [21–24, 29, 43, 44]. *S. aureus* isolates showed 50% resistance to amoxicillin/clavulanic acid. The resistance to the aforementioned antibiotics could be mainly due to excessive use, misuse, and irrational prescription of these medication in Gaza Strip for both hospital and community acquired infections as well as in livestock. Furthermore, the lack of policies for antibiotic prescription, and the commercial availability of antibiotics without a medical doctor prescription in our country worsen the case. In view of the high resistance rates of *S. aureus* to these aforementioned antibiotics, treatment of *S. aureus* infections at our hospitals with these antibiotics may not be effective.

On the other hand, *S. aureus* isolates including both MRSA and MSSA, were mostly susceptible to vancomycin, tetracycline, gentamycin, rifampin and ciprofloxacin. This could be due to the limited prescription and use of these antibiotics in the community in Gaza Strip. So, the full sensitivity to these microbial agents including vancomycin can work perfectly against *S. aureus* and MRSA infections in our community and hospitals as indicated in recent studies in Gaza Strip [9, 10].

Surprisingly, 8 isolates of MRSA (15.7%) and one of MSSA (9.1%) were found to be resistant to vancomycin by disc diffusion test. These findings are alarming for the presence of vancomycin-resistant *S. aureus* (VRSA) among HCWs, which should be addressed in a future larger study in Gaza hospitals.

It is noteworthy saying that our current study was conducted in one hospital, however this hospital (Al-Shifa) is a referral hospital serving the largest population in Gaza strip. Moreover, we realize that our study didn’t involve application of MIC and E-test for vancomycin, as our goal was focusing in assessing the extent of MRSA among HCW rather than assessment of vancomycin sensitivity. MIC and E-test will be applied in future studies involving a larger number of health centers.

## Conclusion

Nasal carriage of MRSA among HCWs is relatively high in Al Shifa, the largest referral hospital in Gaza Strip, particularly among nurses and doctors and in surgery and in internal medicine wards.

These findings highlight the urge for application and adherence to infection control measures that aim at reducing spread of infection by MRSA among susceptible individuals who are highly vulnerable to be infected with MRSA. Active surveillance of MRSA in health care settings is highly recommended.

## Abbreviations

CLSI: Clinical and laboratory standards institute
HCWs: Health care workers
MRSA: Methicillin resistance *Staphylococcus aureus*
MSSA: Methicillin susceptible *Staphylococcus aureus*
PBP2a: Penicillin binding protein 2a
PCR: Polymerase chain reaction
SPSS: Statistical package for the social sciences
VRSA: Vancomycin resistant *Staphylococcus aureus*
